# The effect of FASN inhibition on the growth and metabolism of a cisplatin‐resistant ovarian carcinoma model

**DOI:** 10.1002/ijc.31392

**Published:** 2018-04-01

**Authors:** Efthymia Papaevangelou, Gilberto S. Almeida, Carol Box, Nandita M. deSouza, Yuen‐Li Chung

**Affiliations:** ^1^ Cancer Research UK Cancer Imaging Centre, Division of Radiotherapy and Imaging The Institute of Cancer Research and The Royal Marsden NHS Foundation Trust, 15 Cotswold Road, Belmont Sutton Surrey United Kingdom; ^2^ Department of Surgery and Cancer, Faculty of Medicine, Imperial Centre for Translational & Experimental Medicine (ICTEM) Imperial College London, Hammersmith Hospital Campus London United Kingdom

**Keywords:** FASN, orlistat, ovarian cancer, cisplatin resistance, metabolism, MRS

## Abstract

Overexpression of fatty acid synthase (FASN), a key regulator of the *de novo* synthesis of fatty acids, has been demonstrated in a variety of cancers and is associated with poor prognosis and increased multidrug resistance. Inhibition of FASN with the anti‐obesity drug orlistat has been shown to have significant anti‐tumourigenic effects in many cancers, notably breast and prostate. In our study, we investigated whether FASN inhibition using orlistat is an effective adjunctive treatment for ovarian cancers that have become platinum resistant using a cisplatin‐resistant ovarian tumour xenograft model in mice. Mice were treated with orlistat or cisplatin or a combination and metabolite analysis and histopathology were performed on the tumours *ex vivo*. Orlistat decreased tumour fatty acid metabolism by inhibiting FASN, cisplatin reduced fatty acid β‐oxidation, and combination treatment delayed tumour growth and induced apoptotic and necrotic cell death in cisplatin‐resistant ovarian cancer cells over and above that with either treatment alone. Combination treatment also decreased glutamine metabolism, nucleotide and glutathione biosynthesis and fatty acid β‐oxidation. Our data suggest that orlistat chemosensitised platinum‐resistant ovarian cancer to treatment with platinum and resulted in enhanced efficacy.

## Abstract 2

**What's new?**

Targeting metabolic dependencies of tumor cells has emerged as a promising new therapeutic approach. Here the authors used an approved anti‐obesity drug, orlistat, as an adjuvant treatment for platinum‐resistant ovarian cancer. They show that in mice addition of orlistat re‐sensitises tumors to cisplatin therapy and speculate that the combination of orlistat and cisplatin could be used clinically to treat patients with platinum‐refractory ovarian cancer.

**Abbreviations**
DDIT4: DNA damage‐inducible transcript 4
FA: fatty acid
FASN: fatty acid synthase
MDP: methylene diphosphonic acid
MRS: magnetic resonance spectroscopy
SEM: standard error of the mean
STR: short tandem repeat
TSP: 3‐trimethylsilyl‐2,2,3,3‐tetradeuteropropionate

## Introduction

In cancer cells, the increased genesis of membranes demands an increase in lipogenesis.^1^ This need is met by increased *de novo* fatty acid (FA) biosynthesis, which has been reported in a large number of human malignancies, such as prostate, ovarian and breast.^2^, ^3^, ^4^, ^5^ The process is regulated mainly by fatty acid synthase (FASN) as it catalyses the synthesis of long‐chain fatty acids from acetyl‐CoA, malonyl‐CoA and NADPH precursors.^6^ FASN expression levels are therefore elevated in tumours^7^ and high levels have been associated with cancer progression, aggressiveness, poor prognosis, high risk of disease recurrence^8^, ^9^, ^10^ as well as with drug resistance.^11^, ^12^

Orlistat, a pancreatic lipase inhibitor, approved by the US Food and Drug Administration as an anti‐obesity drug inhibits FASN, and produces antitumour effects in a variety of cancers, including ovarian cancer.^13^, ^14^ It acts as an irreversible inhibitor that forms a covalent adduct with the active serine of the thioesterase domain of FASN^15^ and has been shown to halt cell proliferation in several prostate cancer cell lines *in vitro* and inhibit prostate tumour growth in murine xenografts.^16^ It also reduces proliferation and promotes apoptosis in HER2‐overexpressing breast cancer, ovarian cancer and B16‐F10 mouse metastatic melanoma cells,^17^, ^18^, ^19^ accelerates apoptosis in NUGC‐3 gastric cancer cells *in vitro*, and increases survival rates of gastric tumour‐bearing mice.^20^ However, its effects in overcoming resistance to chemotherapy remain unexplored.

In ovarian cancer, development of platinum resistance signals the onset of difficulties to control disease, so that novel therapeutic approaches are much needed. In our study, we aimed to investigate whether the effects of FASN inhibition by orlistat could overcome cisplatin resistance in ovarian cancer by using it in combination with cisplatin in a cisplatin‐refractory ovarian carcinoma xenograft mouse model and validating the tumour response on histopathology. Magnetic resonance spectroscopy (MRS) was also performed to assess the metabolic changes caused by inhibition of lipid synthesis.

## Materials and Methods

### Cell culture

Cisplatin‐resistant A2780cis (ECACC 93112517) human ovarian carcinoma cells were obtained from European Collection of Authenticated Cell Cultures and maintained in RPMI 1640 culture medium (Sigma‐Aldrich, Dorset, UK) supplemented with 2 mM l‐glutamine and 10% (v/v) fetal bovine serum (Life Technologies, Paisley, UK) and in a humidified atmosphere with 5% CO_2_ at 37°C. To retain resistance, 1 µM cisplatin (Enzo Life Sciences, Exeter, UK) was added to the medium for every other passage. Cells were tested negative for mycoplasma infection using LookOut Mycoplasma PCR (Sigma‐Aldrich), and Short Tandem Repeat (STR) profiling was performed using a GenePrint 10.0 kit (Promega, Southampton, UK) to authenticate the cell line before the *in vivo* experiments.

### Animals and tumours

Animal experiments were performed in accordance with the local ethical review panel, the UK Home Office Animals (Scientific Procedures) Act 1986, and with the UK National Cancer Research Institute Guidelines for the Welfare and Use of Animals in Cancer Research.^21^, ^22^ Female NCr nude mice, 6–8 weeks old, were injected with 5 × 10^6^ A2780cis cells in 0.1 mL serum‐free medium subcutaneously into the right flank. Callipers were used to measure the tumour length (*L*), width (*W*) and depth (*D*) and the volume was calculated assuming an ellipsoid shape using the formula: (π/6) × *L* × *W* × *D*. Approximately 2–3 weeks after cell inoculation, when tumours reached a mean volume of approximately 200 mm^3^, mice were randomly divided into six treatment cohorts (*n* = 6 per cohort).

**Figure 1:**
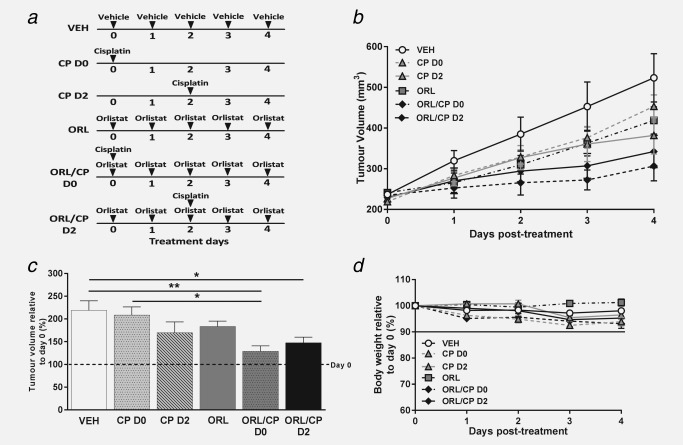
The anti‐tumour effects of orlistat and cisplatin combination therapy in A2780cis human ovarian carcinoma xenografts. (*a*) Diagram of dosing schedule for each mouse cohort (vehicle: saline with 10% ethanol, cisplatin: 5 mg kg^−1^, orlistat: 240 mg kg^−1^). (*b*) Tumour volume changes over the course of treatment and (*c*) tumour volume at Day 4 relative to Day 0 (%) showing an increase in tumour volume in the vehicle‐treated cohort, while drug combination (orlistat and cisplatin) induced significant tumour growth delay (dashed line indicates tumour volume at Day 0). (*d*) Mouse body weight relative to Day 0 of treatment. Data are mean ± 1 SEM for *n* = 6 tumours per group (*p < 0.05, **p < 0.01).

Mice in each cohort where treated intraperitoneally for 5 days with either: (a) a daily dose of 100 µL vehicle (VEH, saline with 10% ethanol), (b) a single dose of 5 mg kg^−1^ cisplatin administered at Day 0 (CP D0) or (c) at Day 2 (CP D2), (d) a daily dose of 240 mg kg^−1^ orlistat (ORL, Cayman Chemical, Michigan, USA) freshly dissolved in vehicle, (e) combination of daily doses of orlistat and single dose of cisplatin at Day 0 (ORL/CP D0) or (f) at Day 2 (ORL/CP D2). A diagram with the treatment doses in each cohort is shown in Figure 1 *a*. Tumours were excised 4 hr after the last orlistat dose at Day 4, cut in half and snap frozen.

### High‐resolution ^1^H and 31P magnetic resonance spectroscopy of tumour extracts

One half of each excised tumour was finely crashed in liquid nitrogen using a mortar and a pestle and extracted by dual phase extraction procedures as previously described.^23^ The water‐soluble extracts were freeze‐dried, reconstituted in 580 µL deuterated water (D_2_O, Sigma Aldrich) and 20 µL of 0.75% sodium 3‐trimethylsilyl‐2,2,3,3‐tetradeuteropropionate (TSP) in D_2_O (Sigma Aldrich) was added to the samples for chemical shift calibration and quantification. 500 μL of the extract solution was transferred to a 5 mm NMR tube and sample pH was adjusted to 7 using perchloric acid or potassium hydroxide. ^1^H MRS of the tumour extracts was performed on a Bruker 500 MHz nuclear magnetic resonance system (Bruker Biospin, Coventry, UK) and spectra were acquired using a pulse and collect NMR sequence with water suppression with 7,500 Hz spectral width, 32 K time domain points, relaxation delay 2.7 sec, 256 scans, temperature 298 K. After ^1^H MRS, EDTA (50 μL, 60 mmol/L) was added to each sample for chelation of metal ions and methylene diphosphonic acid (MDP) (50 μL, 5 mmol/L) was added to samples for 31P chemical shift calibration and quantitation. The pH was again adjusted to 7 and 31P MRS was performed with 12,000 Hz spectral width, 32 K time domain points, relaxation delay 5 sec, 3,000 scans, temperature 298 K.

Lipid extracts were reconstituted in 450 µL deuterated chloroform (Sigma Aldrich) and 150 µL of 0.1% tetramethylsilane (TMS) in deuterated chloroform (Sigma Aldrich) was added to the samples for chemical shift calibration and quantification. ^1^H MRS of the lipid extracts was performed with 7,500 Hz spectral width, 32 K time domain points, relaxation delay 2.7 sec, 256 scans, temperature 298 K.

Spectral processing was carried out using the Bruker Topspin‐2 software package (Bruker Biospin, Coventry, UK) and spectral assignments were based on literature values.^24^, ^25^ Water‐soluble metabolites measured by ^1^H and 31P MRS were quantified relative to TSP or MDP, respectively, and normalised to tumour weight.^23^ Lipid metabolite levels were expressed as peak‐area ratio relative to the reference TMS and normalised to tumour weight.^23^

### Histology

The remaining half of each excised frozen tumour was used for histological analysis. Frozen tumour sections (10 µm thick) were cut axially from two regions for each tumour, one in the centre of the tumour and one 1 mm apart. To assess apoptosis, acetone fixed sections were stained with a rabbit polyclonal anti‐cleaved caspase‐3 antibody (1/20, Abcam, Cambridge, UK) and Alexa Fluor 546 goat anti‐rabbit secondary antibody (1/1,000, Invitrogen, Paisley, UK). To access FASN expression, paraformaldehyde fixed and permeabilised sections were stained with a rabbit monoclonal anti‐FASN antibody [EPR7465] (1/200, Abcam) and Alexa Fluor 488 goat anti‐rabbit secondary antibody (1/1,000, Invitrogen). Non‐immune‐specific rabbit IgG was used in the same concentrations with the anti‐cleaved caspase‐3 antibody and the anti‐FASN antibody, as negative isotype controls. Fluorescent staining was visualised using a motorised scanning stage (Prior Scientific Instruments, Cambridge, UK) attached to a BX51 microscope (Olympus Optical, London, UK) driven by CellP (Soft Imaging System, Munster, Germany) to record digital composite images of whole tumour sections. To quantify the degree of necrosis, sections were also stained with haematoxylin and eosin (H&E), dehydrated through a series of alcohols and xylene and visualised using bright‐field microscopy. Tumour necrotic areas were defined by the presence of microscopic coagulative necrosis with homogeneous clusters and sheets of degenerating and dead cells; necrotic tissue appeared lighter in H&E stained sections due to lack of nuclei. The image analysis software ImageJ^26^ was used for blinded (to the time of treatment) post‐processing of all digital composite tumour images. ROIs encompassing the whole tumour sections were defined and the area of the tumour section with fluorescent staining or with necrosis was expressed as a percentage of the whole tumour section area, as previously described.^27^

### Statistical analysis

Data were analysed using GraphPad Prism 7 (GraphPad Software, Inc., La Jolla, CA). Statistical significance of differences was determined by Student's *t*‐test or one‐way ANOVA with Bonferroni post‐test, with a 5% level of significance. Results are presented as mean ±1 standard error of the mean (SEM).

## Results

### Addition of orlistat to cisplatin chemotherapy caused significant tumour growth delay in cisplatin‐resistant ovarian carcinoma xenografts

A2780cis tumours had a doubling rate of 3.3 ± 0.6 days based on independent tumour volume growth curves for each of the mice used in our study. The mean tumour volume at treatment onset was 227 ± 20 mm^3^. Both cisplatin (CP) and orlistat (ORL) monotherapies induced a small tumour growth delay in comparison with vehicle‐treated tumours, but the delay was significant only when the two drugs were used in combination (Figs. 1 *b* and 1 *c*).

To access whether the effect of cisplatin is depended on whether the tumours have been pre‐exposed to orlistat or not, cisplatin was administered either at the beginning of the treatment (CP D0) or after few days (CP D2) of the mice being treated with orlistat. However, the time point at which cisplatin was administered, either Day 0 or Day 2, did not affect the growth of the combination‐treated tumours. As shown in Figure 1 *c*, the volume of the vehicle‐treated (VEH) tumours increased by 119 ± 51% at Day 4, while the volume of the combination‐treated cohorts increased only by 29 ± 29% and 48 ± 32% respectively for ORL/CP D0 (*p* < 0.01 compared with VEH) and ORL/CP D2 (*p* < 0.05 compared with VEH). Moreover, while cisplatin alone (CP D0) did not delay tumour growth – tumour volume increased by 109 ± 43% – addition of orlistat (ORL/CP D0) to the treatment led to significant growth delay (*p* < 0.05). Despite orlistat and cisplatin combinations having an effect on tumour growth, no significant weight losses were observed in any of the drug‐treated mice compared to vehicle‐treated mice. Weight loss was <5% of the initial body weight across all cohorts of mice (Fig. 1 *d*); indicating that the treatment dosages and schedules used in our study were well tolerated.

### Orlistat treatment led to decreased fatty acid production and glutamine metabolism in A2780cis tumours

**Figure 2:**
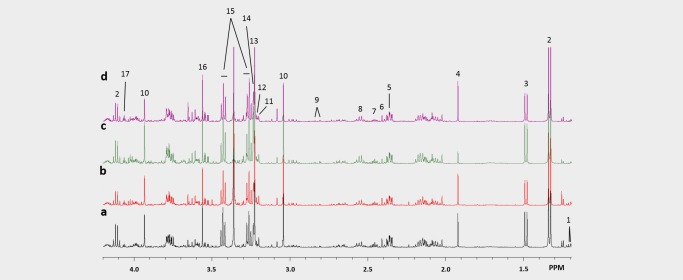
Representative ^1^H MRS spectra of water soluble metabolites in tumour extracts treated with (*a*) vehicle, (*b*) orlistat, (*c*) cisplatin at Day 2 and (*d*) combination at Day 2. Spectral assignments: 1: β‐Hydroxybutyrate; 2: Lactate; 3: Alanine; 4: Acetate; 5: Glutamate; 6: Succinate; 7: Glutamine; 8: Glutathione; 9: Aspartate; 10: Creatine; 11: Carnitine; 12: Choline; 13: Phosphocholine; 14: Glycerophosphocholine; 15: Taurine; 16: Glycine; 17: Myo‐inositol. [Color figure can be viewed at http://wileyonlinelibrary.com]

**Figure 3:**
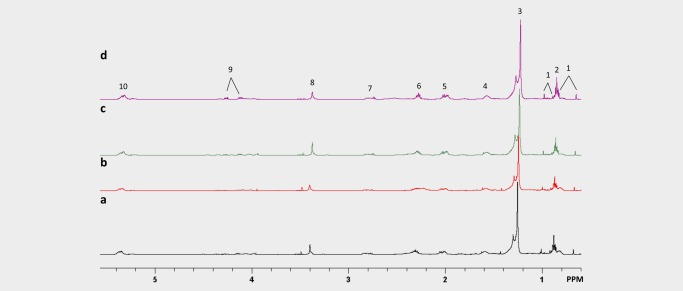
Representative ^1^H MRS spectra of lipid metabolites in tumour extracts from mice treated with (*a*) vehicle, (*b*) orlistat, (*c*) cisplatin at Day 2 and (*d*) combination at Day 2. Spectral assignments: 1: Cholesterol and ester; 2: —CH3 Fatty acid; 3: — (CH2)n— Fatty acid; 4: —CH2—CH2— (CH2)n—; 5: —CH2—CH2—CH=; 6: —CH2—CH2—CO2—; 7: =CH—CH2—CH=; 8: Phosphatidylcholine; 9: Triacylglycerol; 10: —CH=CH—. [Color figure can be viewed at http://wileyonlinelibrary.com]

**Figure 4:**
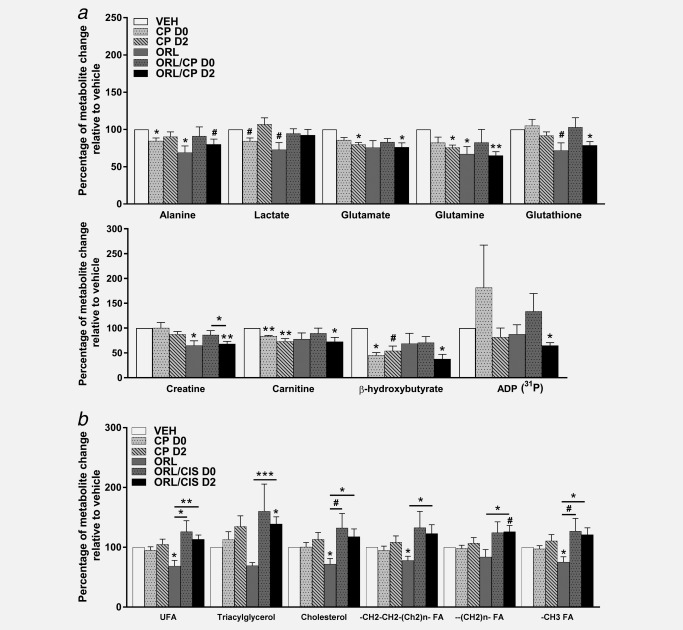
The effect of cisplatin and orlistat treatment in A2780cis xenografts on ^1^H MRS‐detectable metabolites. Changes relative to vehicle (VEH) in the ^1^H MRS‐detectable (^31^P MRS‐detectable for ADP) water‐soluble metabolites (*a*) and lipid metabolites (*b*) of tumour extracts from mice following cisplatin, orlistat or combination therapy. ADP: adenosine diphosphate, UFA: unsaturated fatty acids; FA: fatty acids. Data are mean +1 SEM for *n* = 6 tumours per group (*p ≤ 0.05, **p < 0.01, ***p < 0.001, #*p* < 0.08).

^1^H MRS was performed on Day 4 tumour extracts to analyse levels of water soluble metabolites and lipids (Figs. 2 and 3, respectively). The relative to vehicle percentages of the quantified levels of low molecular weight water‐soluble and lipid metabolites from the A2780cis tumour extracts are shown in Figures 4 *a* and 4 *b*, respectively. Tumours from mice treated with orlistat showed a decrease in alanine (*p* =0.02), glutamine (*p* = 0.04), creatine (*p* = 0.02), glutathione (trend, *p* = 0.06) and also lactate (trend, *p* = 0.06) when compared to vehicle (Fig. 4 *a*). Tumours from cisplatin‐treated mice had lower levels of β‐hydroxybutyrate (*p* = 0.02), alanine (*p* = 0.05), carnitine (*p* = 0.009) and lactate (trend, *p* = 0.07) (D0 cohort), or reduced glutamate (*p* = 0.04), glutamine (*p* = 0.02), carnitine (*p* = 0.008) and β‐hydroxybutyrate (trend, *p* = 0.06) levels (D2 cohort) when compared to the vehicle cohort. Glutamate (*p* = 0.04), glutamine (*p* = 0.004), glutathione (*p* = 0.03), carnitine (*p* = 0.03), creatine (*p* = 0.001), β‐hydroxybutyrate (*p* = 0.01), ADP (*p* = 0.04) and alanine (trend, *p* = 0.07) were decreased in the ORL/CP D2 tumours when compared to VEH group, whereas no significant changes were observed in the ORL/CP D0 cohort (Fig. 4 *a*). Apart from the decrease in ADP in the ORL/CP D2 combination group (*p* = 0.04) when compared to control, no significant changes were seen in the 31P‐MRS spectra of the water‐soluble metabolites between the different treatment cohorts (Supporting Information Fig. S1).

Unsaturated fatty acids (*p* = 0.02), cholesterol (*p* = 0.02), —CH2—CH2— (Ch2)n— fatty acids (*p* = 0.05) and —CH3 fatty acids (*p* = 0.04) were decreased in tumours from orlistat‐treated mice compared to vehicle, whereas triacylglycerol (*p* = 0.05) and —(CH2)n— fatty acids (trend, *p* = 0.06) were elevated in the ORL/CP D2 combination group when compared to vehicle (Fig. 4 *b*). Increased unsaturated fatty acids (*p* = 0.003), triacylglycerol (*p* = 0.0008), cholesterol (*p* = 0.02), —CH2—CH2— (Ch2)n— fatty acids (*p* = 0.04), —(CH2)n— fatty acid (*p* = 0.02) and —CH3 fatty acids (*p* = 0.01) were found in the ORL/CP D2 combination group when compared to orlistat alone (Fig. 4 *b*). Unsaturated fatty acids (*p* = 0.03), cholesterol (trend, *p* = 0.06) and —CH3 fatty acids (trend, *p* = 0.07) were also elevated in ORL/CP D0 combination group when compared to orlistat alone (Fig. 4 *b*).

### Cisplatin‐induced tumour necrosis in A2780cis tumours whereas cisplatin and orlistat combination led to apoptosis

**Figure 5:**
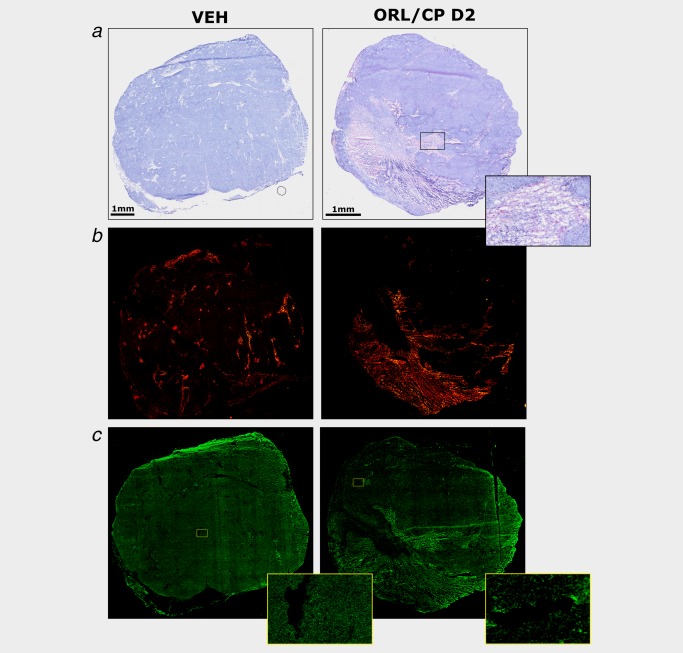
Histological assessment of A2780cis xenografts at Day 4 after treatment with vehicle (VEH) and drug combination (ORL/CP D2). (*a*) Composite images of H&E‐stained sections indicating necrotic regions and magnified image of a necrotic area. (*b*) Composite images from frozen tumour sections stained with the apoptotic marker cleaved caspase‐3 detected using an Alexa‐546‐conjugated secondary antibody that fluoresces red. (*c*) Composite images from whole frozen tumour sections and magnified areas stained with an anti‐FASN antibody detected using an Alexa‐488‐conjugated secondary antibody that fluoresces green. [Color figure can be viewed at http://wileyonlinelibrary.com]

**Figure 6:**
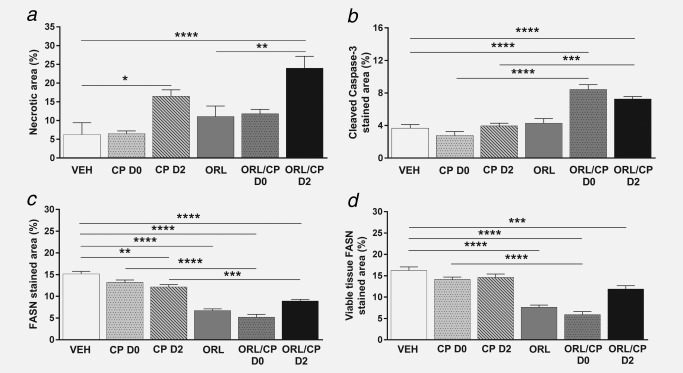
Quantification of histological markers in tumour xenografts. (*a*) Necrotic area, (*b*) cleaved caspase‐3 positive area, (*c*) FASN positive area and (*d*) FASN positive area in viable tissue. Results are means +1 SEM of two sections per tumour for *n* = 6 per group (**p < 0.01, ***p < 0.001, ****p < 0.0001).

Figure 5 shows representative histological sections of excised tumours at Day 4 after treatment with vehicle or combination of cisplatin and orlistat (ORL/CP D2 cohort), stained with H&E (Fig. 5 *a*), cleaved caspase‐3 antibody (Fig. 5 *b*) and FASN antibody (Fig. 5 *c*). Quantification of tumour necrosis using the H&E stained sections at Day 4 after treatment is shown in Figure 6 *a*. Cisplatin administered at Day 2 either as monotherapy or in combination caused a significantly higher degree of necrosis (16 ± 4%, *p* < 0.05 and 24 ± 8%, *p* < 0.0001) compared to vehicle‐treated tumours (6 ± 8%) (Fig. 6 *a*). Cleaved caspase‐3 staining in the cisplatin monotherapy groups (3% for CP D0 and 4% for CP D2) was similar to vehicle controls of 4%, whereas with the addition of orlistat significantly increased cleaved caspase‐3 staining was found in the combination groups (8% in the ORL/CP D0 cohort and 7% in the ORL/CP D2 cohort) when compared to their respective monotherapy (*p* < 0.0001 for ORL/CP D0 and *p* < 0.001 for ORL/CP D2) and with the vehicle controls (*p* < 0.0001 for ORL/CP D0 cohort and *p* < 0.0001 for ORL/CP D2 cohort) (Fig. 6 *b*).

### Treatment with orlistat alone or in combination with cisplatin led to a decrease in FASN expression

FASN staining was 15% in the vehicle cohort, whereas it decreased to 7% when orlistat was given as monotherapy (*p* < 0.0001), and to 5% (*p* < 0.0001) or 9% (*p* < 0.0001) when orlistat was given in combination with cisplatin at Day 0 or Day 2, respectively (Fig. 6 *c*). FASN staining was significantly reduced in the combination groups when compared to their respective cisplatin monotherapy (*p* < 0.0001 for ORL/CP D0 and *p* < 0.001 for ORL/CP D2). A small decrease in FASN staining was also seen in the CP D2 cohort when compared to vehicle controls (*p* < 0.01). However, when the FASN staining was measured only in the viable tissue, to remove the bias of necrotic tissue, no differences in FASN staining where observed in the cisplatin‐treated tumours when compared to vehicle controls (Fig. 6 *d*). The viable tissue fraction was calculated using the necrotic fraction (viable tissue fraction= 1‐necrotic fraction). A significant decrease in FASN staining in the viable tissue fraction was found in tumours treated with the FASN inhibitor orlistat alone or in combination with cisplatin when compared to vehicle controls (*p* < 0.0001 for ORL, ORL/CP D0 and *p* < 0.001 for ORL/CP D2). Moreover, FASN staining was also reduced in the ORL/CP D0‐treated tumours when compared to cisplatin‐treated (CP D0) tumours (*p* < 0.0001).

## Discussion

In our study, we have explored the use of the FASN inhibitor orlistat as a way of overcoming resistance to cisplatin in cisplatin‐refractory ovarian cancer and examined their effects as monotherapies or as a combination, on tumour metabolism. The administered doses of both cisplatin and orlistat were based on previous publications and were well tolerated in our study. Cisplatin was used at its maximum tolerated dose of 5 mg kg^−1^, which has elicited cytotoxic effects in ovarian cancer xenografts.^28^, ^29^, ^30^ Orlistat was used at a daily dose of 240 mg kg^−1^, which has been shown to induce tumour growth delay in human prostate cancer xenografts in nude mice.^16^ Orlistat therapy led to no significant weight loss (<5%) across all treatment groups. This is in agreement with a previous study on mice with subcutaneous human prostate tumours, where the tumourigenic effect of orlistat treatment, at the same daily dose as in our study, was not accompanied by body weight loss.^31^ In contrast, the FASN inhibitors cerulenin and C75, are known to lead to severe decrease in food intake and induction of weight loss in mice.^32^, ^33^, ^34^ Hence, orlistat appears to be a preferable FASN inhibitor in tumours, as it does not cause weight loss, which would be a significant limiting factor in often cachectic cancer patients.

Orlistat has been shown to induce tumour growth delay in preclinical mouse tumour models, such as models of prostate cancer, melanoma, colorectal adenocarcinoma and oral tongue squamous cell carcinoma.^18^, ^31^, ^35^, ^36^ Moreover, using proteomic tools in SKOV3 cells, orlistat has been identified, as a potential inhibitor of human ovarian cancer.^37^ Other FASN inhibitors can also exhibit tumourigenic effects on ovarian cancer models. Pizer *et al*.^38^ has shown that cerulenin can lead to regression of the multiply drug‐resistant OVCAR‐3 human ovarian carcinoma in nude mice. C75 inhibited growth of SKOV3 xenografts grown intraperitoneally in severe combined immunodeficient mice.^34^ In our study, we showed a significant growth delay of cisplatin‐resistant A2780cis tumours following treatment with a combination of orlistat and cisplatin which was greater than that with either drug alone.

Orlistat in combination with cisplatin‐induced apoptosis in our tumours. Previous studies have indicated that orlistat treatment resulted in G1/S stage arrest of cell proliferation and decreased DNA synthesis followed by receptor‐mediated apoptosis via caspase‐8 activation.^39^ Pre‐treatment of MCF‐7 mammary carcinoma cells with orlistat for 24 hr also sensitised the cells to TRAIL‐induced apoptosis. The apoptotic effect of FASN inhibition results from upregulation of the stress response gene DDIT4 (DNA damage‐inducible transcript 4), which negatively regulates the mTOR pathway.^40^ Yang *et al*.^19^ have also shown in ovarian cancer cells that DDIT4 suppressed mTOR to stimulate orlistat‐induced cell death via caspase‐2 activation. In our study, the apoptotic effect of orlistat was significant only when orlistat was used in combination with the cytotoxic agent, cisplatin. Cisplatin treatment on the other hand, induced necrosis either as monotherapy or as combination when administered at Day 2, but not when administered at Day 0. This is probably because the necrotic effect was more profound when the mice were killed 2 days after the cisplatin administration, whilst the tumours had not yet time to recover, rather than 4 days after treatment. It may well be that cisplatin treatment has an initial temporary cytotoxic effect in cisplatin‐resistant A2780cis tumours, and that FASN inhibition acts synergistically to further delay tumour growth and induce apoptosis in the remaining cell population. Moreover, if FASN is involved in the repair process following the initial cellular damage caused by cisplatin treatment by providing lipids for the *de novo* membrane synthesis, FASN inhibition would lead to a shortage of lipids for generating replacement cells.

Recent studies have linked FASN overexpression in cancer with multidrug resistance, which partially explains the association between FASN expression and poor prognosis. FASN blockade reverses the acquired resistance to trastuzumab in breast and ovarian cancer cells^41^, ^42^ and can sensitise breast cancer cells to doxorubicin, docetaxel, paclitaxel and vinorelbine chemotherapy.^11^, ^12^ A combination of cerulenin and 5‐fluorouracil displayed a schedule‐dependent synergistic effect in breast carcinoma cells with maximum efficacy when cells were exposed to 5‐fluorouracil prior to cerulenin.^43^ A proteomic analysis of cisplatin‐resistant mouse mammary tumours identified FASN as a predictive marker for cisplatin resistance; inhibition of FASN sensitised resistant cells to cisplatin.^44^ Inhibiting key metabolic enzymes in the fatty acid synthesis pathway led to significant cell death in cisplatin‐resistant lung cancer cells.^45^ In addition, combination treatment of C75 and cisplatin resulted in growth inhibition of epithelial ovarian carcinoma xenografts in nude mice.^46^ Sequential cerulenin/cisplatin treatment reduced cisplatin's half maximal inhibitory concentration in cisplatin‐resistant ovarian cancer cells, suggesting platinum (re)sensitisation.^47^ In a mouse model of Dalton's lymphoma, cisplatin exhibited a maximal effect on tumour growth retardation when cisplatin was administered following pre‐exposure to orlistat. The same study has also shown that orlistat administration *in vivo* not only resulted in reduced FASN expression and activity, but also reduced the expression of multidrug resistance protein (MDR) and multidrug resistance associated protein‐1 (MRP‐1).^48^ Thus, the study suggested that one of the mechanisms by which orlistat makes tumour cells susceptible to cisplatin cytotoxicity is by inhibiting multidrug resistance regulating molecules. In our study, we have shown that while cisplatin on its own could not slow the growth of A2780cis ovarian tumours, inhibition of FASN with orlistat sensitised the cisplatin‐resistant tumours to cisplatin. The delay in tumour growth was not affected by the time of cisplatin administration despite the differences observed in the tumour metabolic profiles, which were more profound when cisplatin was given at Day 2 rather than Day 0, probably due to the transient nature of metabolic changes.

In our study, we found cisplatin treatment alone leads to decreases in carnitine and β‐hydroxybutyrate in cisplatin‐resistant ovarian tumours, indicating that β‐oxidation of fatty acids is impaired following cisplatin treatment. Carnitine is a non‐essential amino acid involved in the transport of fatty acids across the mitochondrial membrane for β‐oxidation.^49^ β‐hydroxybutyrate is a ketone body and a product of fatty acid oxidation. Therefore, a reduced level of carnitine in tumours could lead to a subsequent reduction in β‐oxidation of fatty acids and ketone body production. Our finding is consistent with a previous report in which l‐carnitine was used to inhibit cisplatin‐induced injury in kidney and small intestine where there are very high level of carnitine transporters.^50^ Decreased glutamine and glutamate levels were observed only in tumours treated with cisplatin for 2 days (CP D2) and not after 4 days (CP D0), this may indicate that the change in glutamine metabolism is an acute effect, which normalises after a longer period of time.

Treatment of orlistat alone led to decreased cholesterol, saturated and unsaturated fatty acids in tumours together with reduced FASN expression, confirming the mechanism of drug action. Previous studies have also seen a reduction in FASN expression in mouse metastatic melanoma cells and in human glioblastoma cells.^18^, ^51^ This could be either due to decreased expression in a translational level or increased degradation of the FASN protein. When orlistat was combined with cisplatin (at either time point), MRS‐measured lipid levels in the tumours were increased when compared to orlistat alone despite the FASN expression remaining suppressed. This is due to the fact that the combination treatment caused tumours to undergo apoptosis (measured by cleaved caspase‐3 staining), and increases in lipids were previously reported as one of the metabolic features for apoptosis.^52^, ^53^, ^54^ As orlistat reduced tumour lipids on one hand and apoptosis caused elevation of lipid levels on the other, this could lead to lipid levels becoming normalised as observed in the orlistat and cisplatin combination treated‐tumours compared to the vehicle‐treated tumours. Altered lipid metabolism is a common consequence of apoptosis and various apoptosis‐inducing treatments are known to increased NMR‐visible lipid signals. The majority of the lipid signals is produced by lipids located in cytoplasmic lipid droplets/bodies.^55^ The lipid body formation in apoptosis could be due to increased catabolism of membrane phospholipids, which produces free fatty acids that are converted to triacylglycerols and stored in lipid bodies. Another potential source of mobile lipids could by the breakdown of mitochondrial membranes or the inhibition of phospholipid biosynthesis, which leads to accumulation of diacylglycerols and triacylglycerols.^56^, ^57^

Glutamine is required to sustain cancer cell growth and for cell survival under stress.^58^ It is transported into cells by transporters such as SLC1A5, and it is then converted to glutamate by the enzyme glutaminase. Glutamate is then further metabolised to α‐ketoglutarate in the TCA cycle in the mitochondria to be further utilised for protein, nucleotide and lipid synthesis. Along with reduced lipid metabolites, orlistat treatment alone also caused decreases in alanine, glutamine, creatine and lactate with unchanged glucose level when compared to vehicle controls, suggesting that glutamine metabolism/glutaminolysis is downregulated.^59^ The unchanged levels of β‐hydroxybutyrate and carnitine in orlistat‐treated tumours when compared to vehicle controls indicates β‐oxidation of fatty acids is unaffected by orlistat as a single agent. The tumours could continue to undergo β‐oxidation of fatty acids to maintain tumour bioenergetics (ATP level), despite a reduction in glutamine metabolism/glutaminolysis following orlistat treatment.

Reductions in glutamate, glutamine, glutathione, carnitine, creatine, β‐hydroxybutyrate and ADP were found in tumours treated with the combination of orlistat and cisplatin (ORL/CP D2), indicating that fatty‐acid β‐oxidation and glutamine metabolism are compromised in these tumours which could result in reduced nucleotide and glutathione production. The observed induction of apoptosis in the combined orlistat and cisplatin‐treated groups could be the result of reduced nucleotides and/or glutathione synthesis. Glutathione is an important antioxidant and its depletion could cause an increase in reactive oxygen species and oxidative damage, which could lead to induction of apoptosis. Our data are consistent with a recent study showing the platinum‐resistant ovarian cancer cells can be re‐sensitised to platinum treatment by targeting glutamine metabolism.^60^ Our study has shown that combining cisplatin treatment with FASN inhibition caused downregulation of glutamine metabolism/glutaminolysis and β‐oxidation, which then led to reduction in nucleotides and glutathione synthesis.

In conclusion, we have demonstrated that a combination of cisplatin and orlistat resulted in enhanced treatment efficacy in cisplatin‐resistant ovarian cancer with increased tumour growth delay and induction of apoptotic and necrotic cell death. A combination of these two drugs also led to decreases in glutamine metabolism/glutaminolysis, biosynthesis of nucleotides and glutathione and fatty acid β‐oxidation. The combined effects of these metabolic changes may play a role in the improved efficacy. Hence, FASN inhibitors, such as orlistat, are promising anticancer agents that lead to chemosensitisation and enhanced efficacy when used as part of a combination treatment regime.

## Supporting information

Supporting Information
