# Supplementary material for: The effect of FASN inhibition on the growth and metabolism of a cisplatin‐resistant ovarian carcinoma model
Source: Int J Cancer. 2018 Apr 1;143(4):992–1002. doi: 10.1002/ijc.31392 (PMC6055739; doi:10.1002/ijc.31392)
Supplement: Supplementary file 1 — Supporting Information [file IJC-143-992-s001.pdf]

Supplementary online material for "The effect of FASN inhibition on the growth and metabolism of a cisplatin-resistant ovarian"

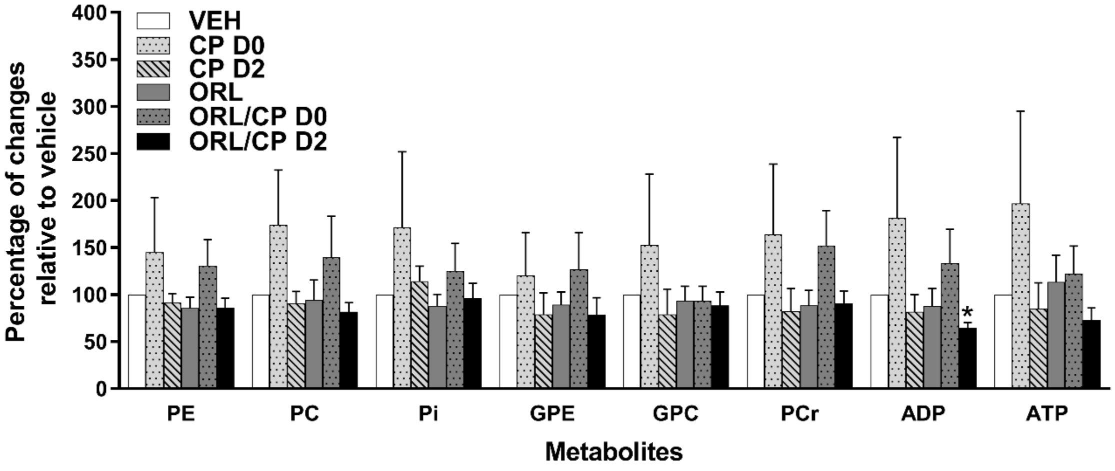

Supplementary Figure 1 The effect of cisplatin and orlistat treatment in A2780cis xenografts on ^{31}P MRS-detectable metabolites. Changes relative to vehicle (VEH) in the ^{31}P MRS-detectable water-soluble metabolites of tumour extracts from mice following cisplatin, orlistat or combination therapy. PE: phosphoethanolamine, PC: phosphocholine, Pi: inorganic phosphate, GPE: glycerophosphoethanolamine, GPC: glycerophosphocholine, PCr: phosphocreatine. Data are mean +1 SEM for $n = 6$ tumours per group (*$P < 0.05$).
